# Gold Nanoparticle Mediated Multi-Modal CT Imaging of Hsp70 Membrane-Positive Tumors

**DOI:** 10.3390/cancers12051331

**Published:** 2020-05-22

**Authors:** Melanie A. Kimm, Maxim Shevtsov, Caroline Werner, Wolfgang Sievert, Wu Zhiyuan, Oliver Schoppe, Bjoern H. Menze, Ernst J. Rummeny, Roland Proksa, Olga Bystrova, Marina Martynova, Gabriele Multhoff, Stefan Stangl

**Affiliations:** 1 Department of Diagnostic and Interventional Radiology, Klinikum rechts der Isar der Technischen Universität München, 81675 Munich, Germany; Melanie.Kimm@tum.de (M.A.K.); Ernst.Rummeny@tum.de (E.J.R.); 2 Central Institute for Translational Cancer Research (TranslaTUM), Klinikum rechts der Isar der Technischen Universität München, 81675 Munich, Germany; Maxim.Shevtsov@tum.de (M.S.); c.werner@tum.de (C.W.); Wolfgang.Sievert@tum.de (W.S.); zhiyuan2012.wu@tum.de (W.Z.); Oliver.Schoppe@tum.de (O.S.); Bjoern.Menze@tum.de (B.H.M.); Gabriele.Multhoff@tum.de (G.M.); 3 Pavlov First Saint Petersburg State Medical University, 197022 St. Petersburg, Russia; 4 Institute of Cytology of the Russian Academy of Sciences (RAS), 194064 St. Petersburg, Russia; o3608338@gmail.com (O.B.); mgmart14@mail.ru (M.M.); 5 Institute for Advanced Studies, Department of Informatics, Technical University of Munich, 85748 Garching, Germany; 6 Philips GmbH Innovative Technologies, Research Laboratories, 22335 Hamburg, Germany; roland.proksa@philips.com

**Keywords:** gold nanoparticle, heat shock protein 70, molecular imaging, biomarker, spectral-CT

## Abstract

Imaging techniques such as computed tomographies (CT) play a major role in clinical imaging and diagnosis of malignant lesions. In recent years, metal nanoparticle platforms enabled effective payload delivery for several imaging techniques. Due to the possibility of surface modification, metal nanoparticles are predestined to facilitate molecular tumor targeting. In this work, we demonstrate the feasibility of anti-plasma membrane Heat shock protein 70 (Hsp70) antibody functionalized gold nanoparticles (cmHsp70.1-AuNPs) for tumor-specific multimodal imaging. Membrane-associated Hsp70 is exclusively presented on the plasma membrane of malignant cells of multiple tumor entities but not on corresponding normal cells, predestining this target for a tumor-selective in vivo imaging. In vitro microscopic analysis revealed the presence of cmHsp70.1-AuNPs in the cytosol of tumor cell lines after internalization via the endo-lysosomal pathway. In preclinical models, the biodistribution as well as the intratumoral enrichment of AuNPs were examined 24 h after i.v. injection in tumor-bearing mice. In parallel to spectral CT analysis, histological analysis confirmed the presence of AuNPs within tumor cells. In contrast to control AuNPs, a significant enrichment of cmHsp70.1-AuNPs has been detected selectively inside tumor cells in different tumor mouse models. Furthermore, a machine-learning approach was developed to analyze AuNP accumulations in tumor tissues and organs. In summary, utilizing mHsp70 on tumor cells as a target for the guidance of cmHsp70.1-AuNPs facilitates an enrichment and uniform distribution of nanoparticles in mHsp70-expressing tumor cells that enables various microscopic imaging techniques and spectral-CT-based tumor delineation in vivo.

## 1. Introduction

Detection of all malignant tumor cells in a patients’ body is a prerequisite for a successful therapy outcome. In established clinical routine, combined positron emission tomography (PET)/computed tomography (CT) imaging is commonly used for tumors exceeding 0.5–1 cm^3^. For standard clinical PET imaging, ^18^F-glucose is often used as a PET tracer. However, glucose-based PET/CT imaging faces several disadvantages, such as false-positive and/or false-negative signals, e.g., triggered by the fact that only metabolically active but not resting cells can be visualized, the low tumor-to-background contrast, and the relatively low resolution of the technique [1]. With improved settings, a spatial resolution of 2 mm is technically feasible, as demonstrated in patients with prostate cancer [2].

The introduction of gold nanoparticles (AuNP)-based contrast agents added a new value to imaging techniques. Functionalization of novel metal-based nanoparticles with tumor-specific antibodies [3] and their utilization in imaging techniques such as photoacoustic tomography or CT combine the advantages of a molecular, tumor-specific imaging with the unique attributes of AuNP in clinical imaging. For instance, spectral-CT technology employs a photon-counting detector which registers the interactions of individual photons, creating a certain energy spectrum which can subsequently be converted into a color image. At the same time, a nonspectral attenuation image can be acquired. This combination allows a precise spatial information with a high resolution. With the emergence of clinical spectral-CT scanners, the need of tumor-specific contrast agents further increased. In this setting, AuNPs might play a crucial role as the energy-dependent X-ray attenuation properties (K-edge at 80.7 keV) allow an excellent separation from calcium (K-edge at 4 keV) and iodine (K-edge at 33.2 keV) [4].

For in vivo application, it is also essential that the applied nanoparticles are nontoxic, biodegradable or inert, and easily transportable in the blood and/or lymphatic system. Biocompatible camouflage of the NP surface is a prerequisite to avoid immediate uptake by macrophages. Small AuNPs (<100 nm) demonstrated to be beneficial for utilization in clinical applications [5,6,7]. Apart from the formulation of AuNPs, tumor imaging with nanoparticle-based contrast agents can be further improved and specified by functionalization with antibodies targeting tumor-specific, membrane-bound biomarkers. For an improved signal-to-background ratio and a high tumor specificity, candidate markers should be selectively expressed on tumor cells while being absent on healthy cells. Membrane-bound Heat shock protein 70 (Hsp70, Hsp70-1, HspA1A, #3303) has been found to fulfill these criteria, exhibiting a remarkable tumor-specific targeting capability [8,9]. In addition to the physiological, cytosolic expression in all nucleated cells, Hsp70 is also found on the plasma membrane of malignantly transformed cells. Upon stress, this molecular chaperone has been found to be increased in the cytosol and on the plasma membrane of different murine and human tumor cells. Screening of tumor biopsies of over 1200 patients has shown that the majority of the primarily diagnosed carcinoma samples but none of the tested corresponding normal tissues exhibited a membrane Hsp70-positive phenotype [10,11,12,13]. After therapy of tumors with standard regimens, such as radiotherapy or chemotherapy, the membrane expression density of Hsp70 on tumor cells is increased [14], which in turn further improves targeting of membrane Hsp70-positive tumors after standard therapies. Furthermore, an upregulated membrane Hsp70 density could be detected on relapse tumors and metastases compared to primary tumors. In multiple studies, the malignancy of tumors correlates positively with the Hsp70 expression density in the cytoplasm and on the plasma membrane [8,11].

For a specific in vivo tumor targeting which is mediated by membrane-bound Hsp70, we developed the membrane Hsp70-specific antibody cmHsp70.1 [9,15]. To utilize the beneficial features of targeting membrane Hsp70 with the imaging capabilities of gold as a contrast agent, we developed an AuNP formulation, functionalized with cmHsp70.1 monoclonal antibody to target membrane-bound Hsp70 on tumor cells in vitro and in vivo. In previous studies, we could demonstrate a rapid and specific binding, uptake, and internalization of cmHsp70.1-AuNPs into tumor cells in vitro, leading to a high intracellular accumulation. Furthermore, following incubation of viable tumor cells with cmHsp70.1-AuNPs, no severe toxic side effects were observed up to a concentration of 10 µg/mL [16].

## 2. Results

### 2.1. Functionalization of AuNPs with cmHsp70.1 Antibody

**Figure 1:**
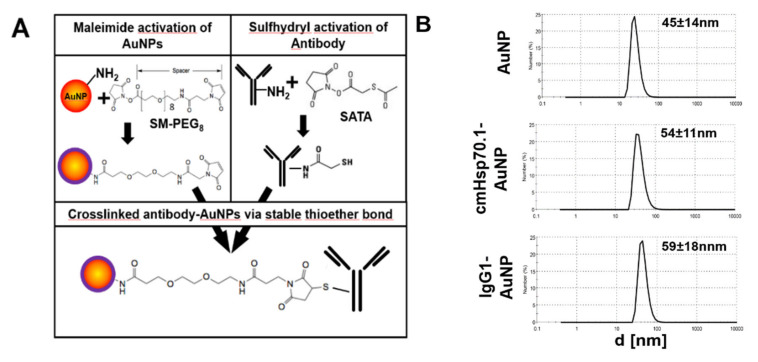
Antibody conjugation of gold nanoparticles (AuNPs) and characterization: (**A**) Coupling reaction of maleimide-activated AuNPs and sulfhydryl-activated monoclonal antibodies. (**B**) Size distribution of the differently functionalized AuNPs, given in size by number histograms.

**Figure A1:**
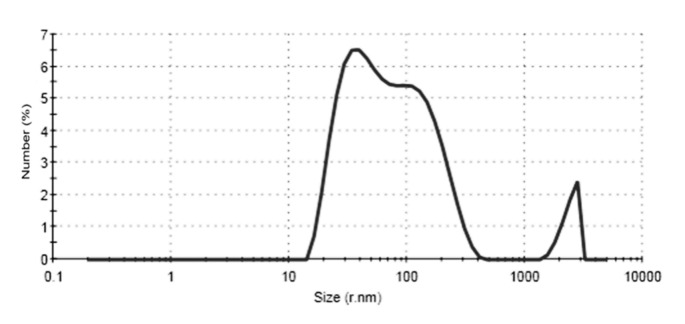
Size distribution of cmHsp70.1-AuNPs after 2 weeks at 4 °C: Size distribution is given in size by number histogram.

**Figure A2:**
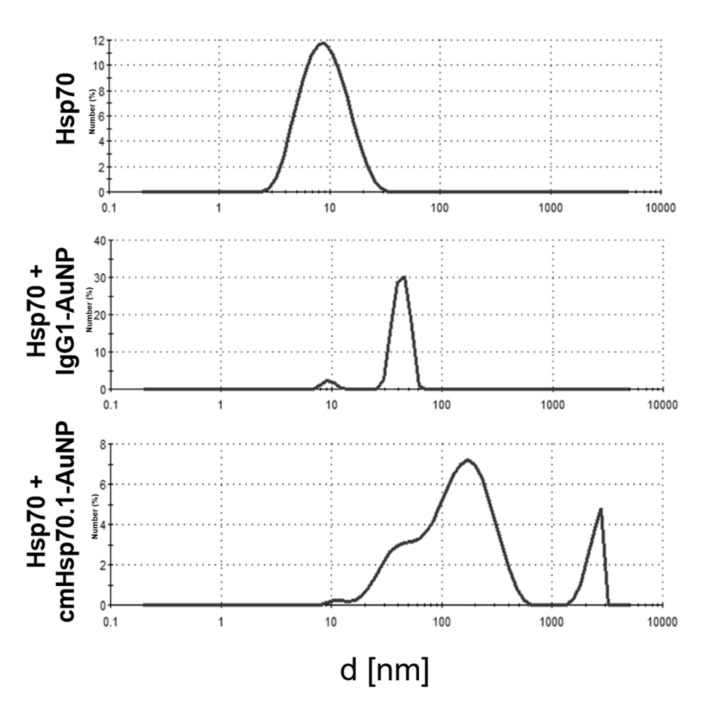
Size distribution of recombinant Hsp70 (upper panel) as well as IgG1-AuNPs (middle panel) and cmHsp70.1-AuNPs (lower panel) following incubation with recombinant Hsp70 for 4 h: Size distribution is given in size by number histograms.

For the coupling of mouse IgG1 isotype-matched or cmHsp70.1 antibody to AuNPs, we used a standard maleimide coupling reaction (Figure 1A). The size of the mean hydrodynamic diameter of unconjugated AuNPs was determined to be 45 ± 14 nm (Figure 1B, top panel). The sizes of cmHsp70.1 antibody-conjugated cmHsp70-AuNPs were 54 ± 11 nm (Figure 1B, middle panel) and 59 ± 18 nm for IgG1 isotype-matched control antibody-conjugated gold nanoparticles (IgG1-AuNPs) (Figure 1B, bottom panel). No self-aggregation was observed in aliquotes of the conjugated as well as the unconjugated AuNPs in phosphate buffered saline (PBS) at 37 °C during 24 h. After 4 weeks of storage at 4 °C, self-aggregation of the particles was observed. An exemplary size distribution histogram is given in Figure A1. To determine the Hsp70-specific binding capacity of cmHsp70.1-AuNPs, we performed analysis of the interaction of cmHsp70.1-AuNPs as well as IgG-AuNPs with recombinant human Hsp70, using an agglomeration assay, as described by Shevtsov et al. [17]. Following a 4 h incubation period with recombinant Hsp70, the size of the formed clusters, as determined by dynamic light scattering (DLS), was larger after incubation with cmHsp70.1-AuNPs (mean event sizes: 152 nm and 2420 nm) than with IgG1-AuNPs (52.9 nm), indicating the formation of ligand-mediated agglumerates. The hydrodynamic diameter of recombinant Hsp70 was determined to be 9.7 nm (Figure A2).

### 2.2. Uptake and Internalization of Functionalized AuNPs in Tumor Cells In Vitro

**Figure 2:**
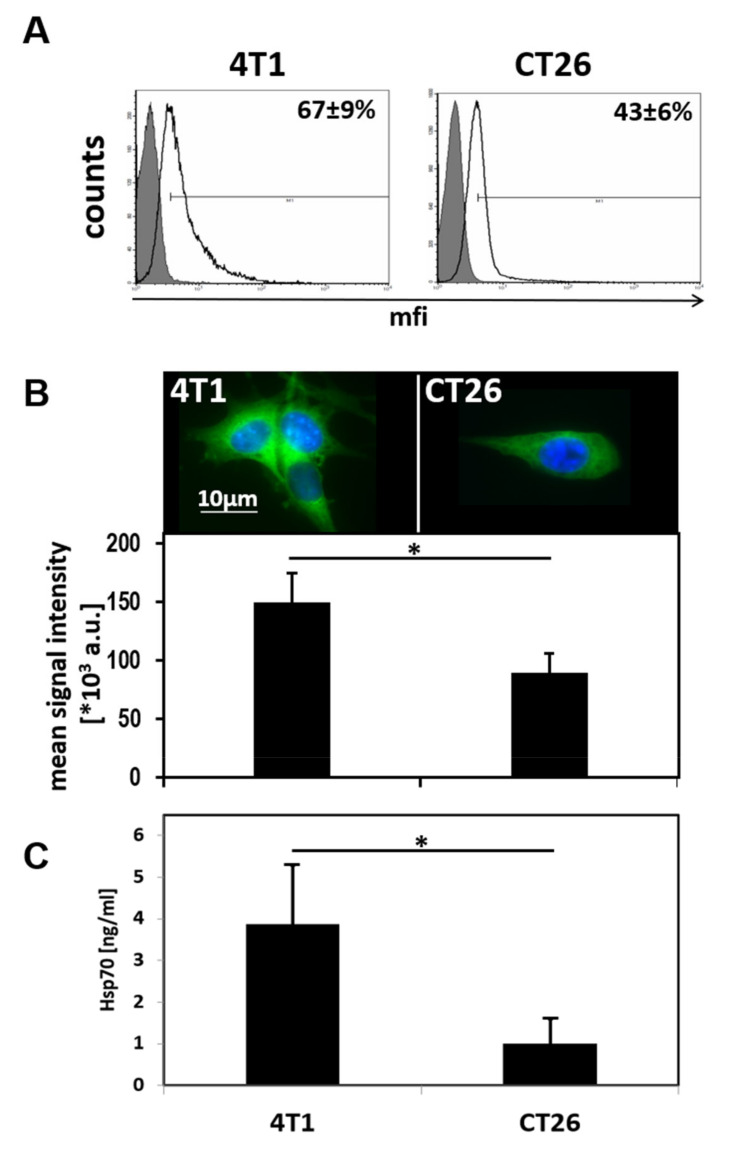
Quantification of Hsp70 in target tumor cell lines: (**A**) Plasma-membrane bound Hsp70 on 4T1 (left) and CT26 (right) cell lines, as determined by flow cytometry. (**B**) Quantitative staining of total Hsp70 in 4T1 and CT26 cells (upper panel) and quantification (lower panel), as determined by an in-cell ELISA technique. (**C**) Quantification of total Hsp70 in whole cell lysates of 4T1 and CT26 cell lines, as determined by an Hsp70 sandwich ELISA. * *p* < 0.05.

**Figure 3:**
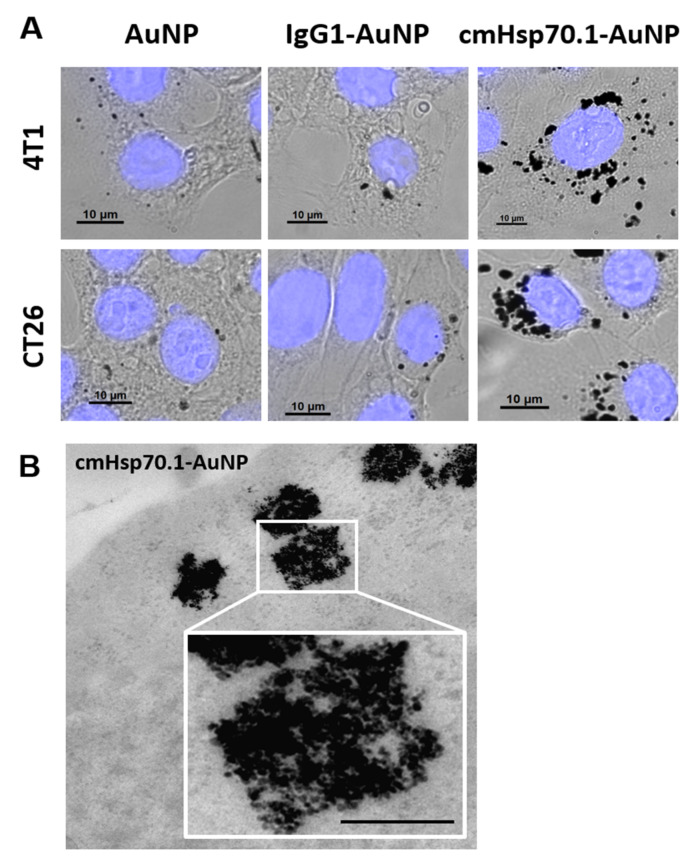
Uptake of AuNPs in tumor cells: (**A**) Intracellular accumulation of blank AuNPs (left), IgG1-AuNPs (middle), and cmHsp70.1-AuNPs (right) in 4T1 (upper panel) and CT26 (lower panel) cells. (**B**) TEM image of intracellular accumulations in 4T1 cells. Magnification is of the indicated area (white box). Scale bar, 1 µm.

To verify the binding capacities and specific uptake of cmHsp70.1-AuNPs in comparison to control nanoparticles (AuNPs and IgG1-AuNPs) in vitro, we performed binding tests on viable, membrane Hsp70-positive tumor cells. To determine the density of the target antigen, the cell lines 4T1 and CT26 were analyzed for their membrane and cytosolic expression of Hsp70. The Hsp70 high expressing cell line 4T1 showed a membrane Hsp70-positive phenotype on 67% ± 9% of the cells, whereas CT26 cells showed a positive phenotype on 43% ± 6% of the cells (Figure 2A). For determination of the total Hsp70 density in the cell lines, an in-cell ELISA technique was established for cells grown in chamber slides. The Hsp70 mean signal intensity in 4T1 and CT26 cell lines were 150.11 × 10^3^ ± 24.92 × 10^3^ a.u. and 89.39 × 10^3^ ± 17.19 × 10^3^ a.u., respectively (Figure 2B). These data were verified by a sandwich ELISA of cell lysates derived from 10 × 10^6^ cells of each cell line, resulting in Hsp70 contents of 4.28 ± 1.74 ng/mL and 1.17 ± 0.72 ng/mL for 4T1 and CT26 cells, respectively (Figure 2C). Subsequently, both cell lines were incubated with the three different types of AuNPs for 24 h at 37 °C to mimic the uptake in living cells. In both cell lines, the content of AuNPs was visualized by brightfield and electron microscopy (Figure 3). Compared to blank AuNPs (Figure 3A, left panel) and IgG1-AuNPs (Figure 3A, middle panel), which showed minor cytosolic uptake, cmHsp70.1-AuNPs displayed the strongest accumulation in both 4T1 and CT26 cells (Figure 3A, right panel). In transmission electron microscopy (TEM), the cmHsp70.1-AuNPs have been found to accumulate in intracellular vesicles of both 4T1 and CT26 cells 24 h after incubation. A representative image of cmHsp70.1-AuNPs in 4T1 cells is shown in Figure 3B.

### 2.3. Accumulation of Functionalized AuNPs in Tumors In Vivo

**Figure 4:**
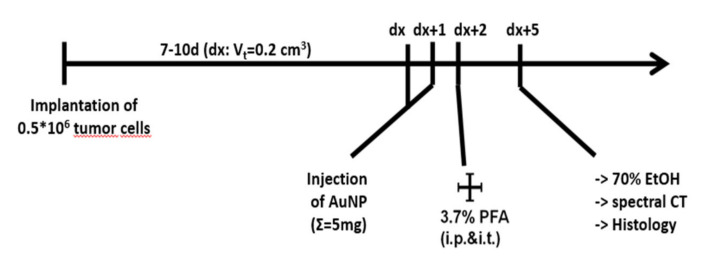
Timeline of the in vivo experiments.

To investigate the specificity and sensitivity of cmHsp70.1-conjugated AuNPs to target tumors in vivo, syngeneic tumor models in Balb/c mice were established. Animals were injected orthotopically (o.t.) with 4T1 and subcutaneously with CT26 tumor cells, respectively. When tumors reached a size of 200 mm^3^, two times 2.5 mg of AuNPs of each group (AuNP, IgG1-AuNP, and cmHsp70.1-AuNP) were injected i.v. consecutively at an interval of 24 h (Figure 4).

**Figure 5:**
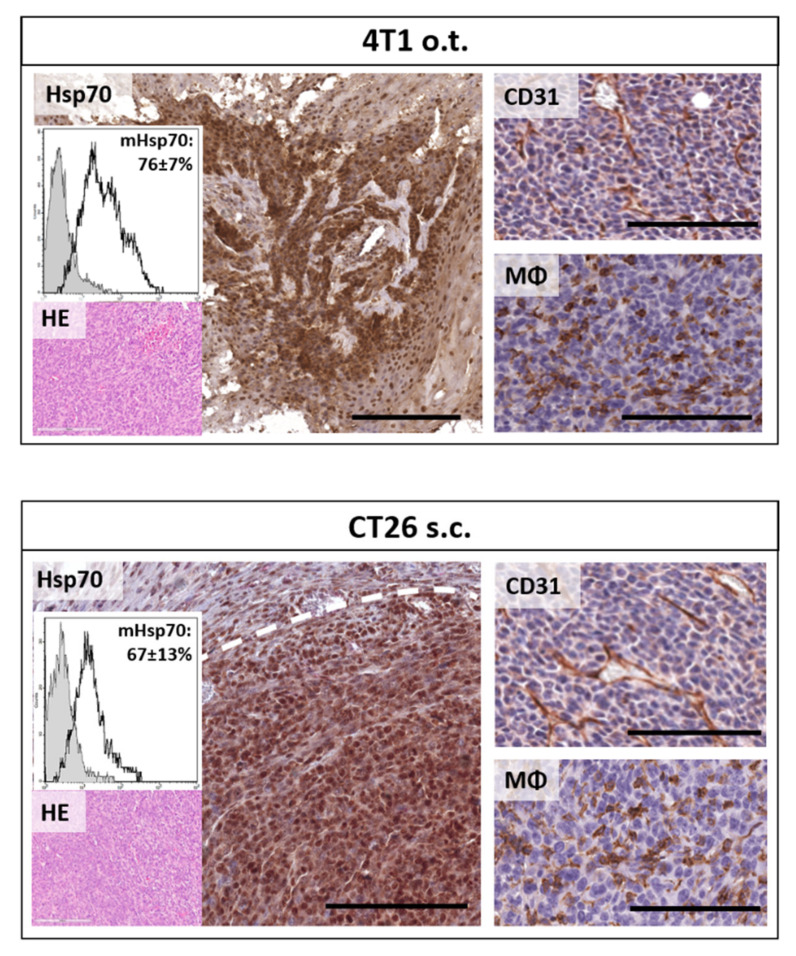
Tumor characterization: Orthotopic (o.t.) 4T1 (upper panel) and subcutaneous (s.c.) CT26 (lower panel) tumors were analyzed with regard to membrane (flow cytometry analysis, upper inlay) and overall (immunohistochemistry, IHC) Hsp70 expression as well as their content of CD31 positive vessels (IHC and CD31) and the infiltration of macrophages (MΦ, IHC, and F4/80). Hematoxylin & Eosin (H&E) was used as an overview stain (lower inlay). Scale bars, 200 µm (Hsp70) and 100 µm (CD31 and MΦ).

Twenty-four hours after the second injection, mice were euthanized and fixed and subsequently imaged using spectral-CT or tumors and organs were directly applied to histological analysis. In parallel, single cell suspensions of tumors of both models have been analyzed for their plasma membrane Hsp70 status. To characterize the tumor models regarding the main features which determine the accumulation of molecular functionalized contrast agents in vivo, tumors were histologically analyzed for the target antigen content (Hsp70) in the cytosol as well as on the plasma membrane. Their vascularization status (CD31) and the presence of tumor infiltrating macrophages (F4/80) have been investigated as well. Both tumor types, 4T1 (o.t.) and CT26 (s.c.), displayed similar vascularization and infiltration of macrophages, indicating comparable effects on the NP input through these routes. Immunohistological Hsp70 staining revealed a strong expression in both tumor models, featuring cytosolic as well as nuclear Hsp70 expression. However, 4T1 tumors showed a more patterned architecture of the Hsp70 density. To investigate the membrane Hsp70 status of the tumors in vivo, a single cell suspension of freshly dissected tumors was investigated. With 76% ± 7% and 67% ± 13% membrane Hsp70-positive viable tumor cells, 4T1 and CT26 tumors grown in vivo, respectively, showed a slightly higher Hsp70 expression density compared to the in vitro cultured cells. However, the increased width of the cytometric data, as given in histograms, indicates an increased heterogeneity in the membrane Hsp70 expression pattern in in vivo grown tumors (Figure 5).

**Figure 6:**
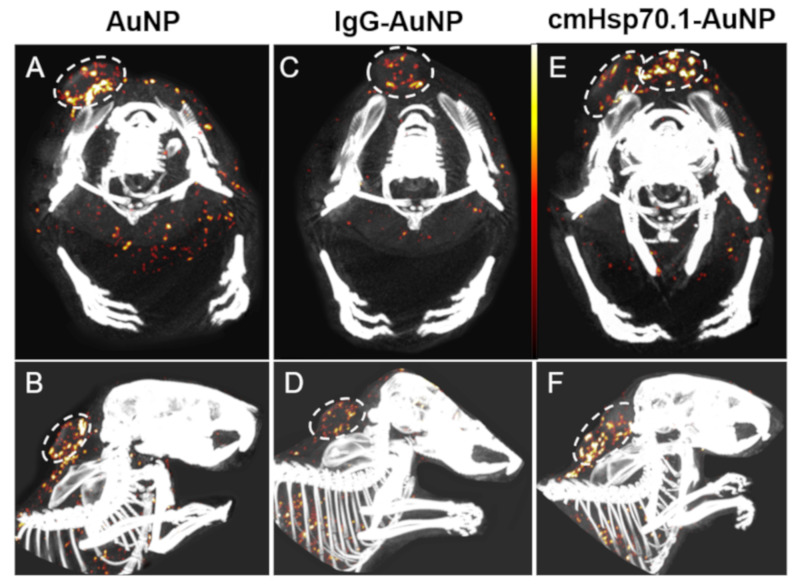
Tumor detection using spectral-CT. Upper Row (**A**,**C**,**E**): axial view, bottom row (**B**,**D**,**F**): sagital view. AuNP amounts are pseudo-coloured from black (6.5 mg/mL) over red (10 mg/mL) to white (13.5 mg/mL). (**A**,**B**) spectral CT-views of mice injected with AuNPs; (**C**,**D**) spectral CT-views of mice injected with IgG-AuNPs; (**E**,**F**) spectral CT-views of mice injected with cmHsp70.1-AuNPs.

In a next step, we investigated the feasibility of cmHsp70.1-AuNPs as a contrast agent in a first cohort of tumor-bearing mice. For this pilot study, three mice with subcutaneous CT26 tumors underwent spectral CT imaging postmortem. Each mouse had received one type of AuNP 24 h before sacrifice. Interestingly, in all animals, we were able to detect AuNPs in the tumors with the highest density of nanoparticles in the tumor periphery. Nevertheless, we also noticed some striking differences. The mouse which was treated with IgG1-AuNPs presented the lowest content of AuNPs inside the tumor (3.3 µg Au/mm^3^ tumor) with a very low accumulation of particles within the tumor center (Figure 6C,D). In the mouse which was treated with unconjugated AuNPs, 4.3 µg Au/mm^3^ was detected in total but mainly at the periphery of the tumor, with considerably less AuNPs in the tumor center (Figure 6A,B). In case of an injection of cmHsp70.1-AuNPs, 4.4 µg Au/mm^3^ was found inside the tumor. Despite similar amounts of different AuNPs in the tumor area, the distribution pattern of the AuNP formulation differed drastically. CmHsp70.1-AuNPs were found to be located in the tumor periphery and highly dispersed in the tumor center (Figure 6E,F).

**Figure 7:**
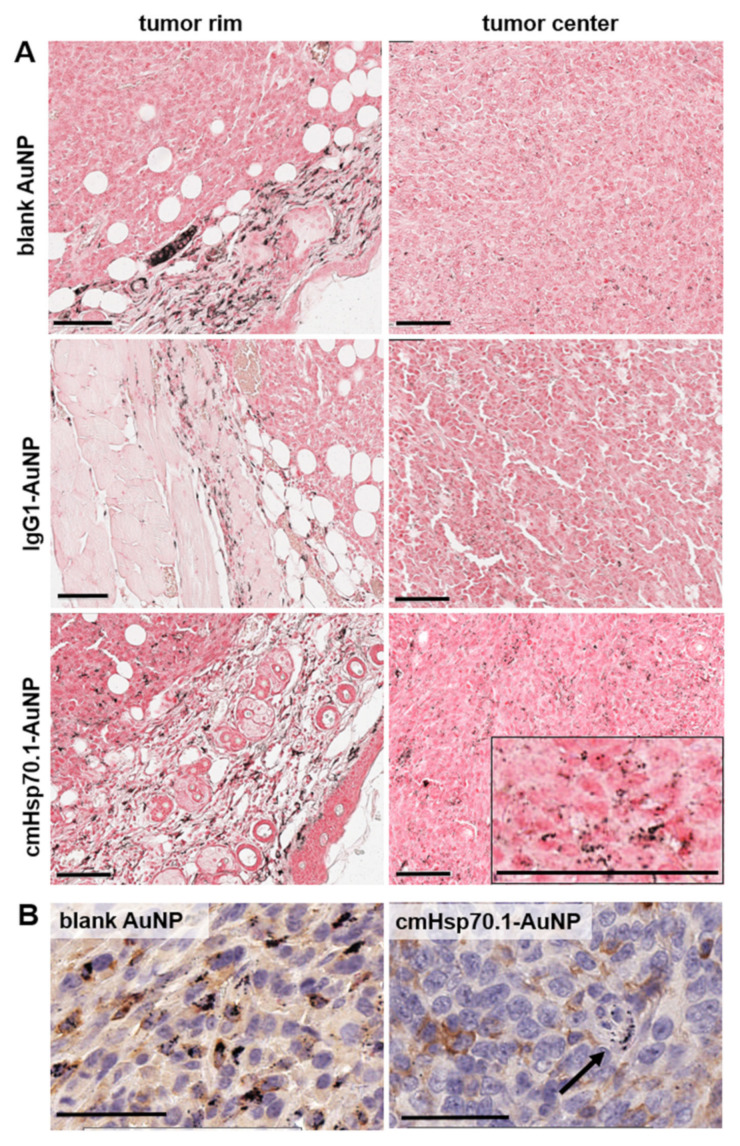
AuNP uptake and distribution in CT26 tumors: (**A**) Silver enhancement of AuNPs. Upper row: blank AuNPs, middle row: IgG1-AuNPs, bottom row: cmHsp70.1-AuNPs. Left: Region of Interest (ROI) at tumor rim area, right: ROI set to tumor center. Scale bars, 100 µm. (**B**) Double staining of Macrophages (F4/80, brown) and silver enhancement of AuNPs (black). Arrow: single-positive cells for AuNPs. Scale bars: 5 µm.

The spectral CT-based biodistribution analysis detected a high accumulation of AuNPs in the spleen, lower amounts in the liver, and very low amounts of AuNPs in the lung. The other organs did not exhibit concentrations which were high enough to generate a signal in the spectral CT measurements. However, to prove data for statistical significance, the number of mice within the study groups have to be increased to relevant numbers in future experiments. The tumors were further analyzed for their gold content by histology (Figure 7).

Sections were treated with silver enhancement to visualize AuNPs by light microscopy (Figure 7A). As revealed by spectral-CT, histological analysis confirmed the varying intratumoral distribution of the three groups of AuNPs. In tumors of mice which were treated with cmHsp70.1-AuNPs, a rather homologous distribution of NPs was observed throughout the whole tumor volume compared to the blank AuNPs or IgG1-AuNPs. Notably, the enrichment of AuNPs at the tumor rim following i.v. injection of blank AuNPs was most likely due to F4/80 positive monocyte/macrophage lineages with internalized AuNPs (Figure 7B, left). In contrast, next to the payload introduction via macrophages, cmHsp70.1-AuNPs were also found in large amounts in F4/80-negative tumor cells. Consequently, an extended accumulation of the cmHsp70.1-AuNPs was also detected in the tumor center. In this region, we also identfied less AuNP containing macrophages (Figure 7B, right). IgG1-AuNPs showed the lowest accumulation in all regions of the tumor (Figure 7A, middle). These NPs, besides their lack of targeting ability, exhibited equal biocompatibility to that of cmHsp70.1-AuNPs due to the conjugation of the murine IgG1 antibody. Consequently, these IgG1NPs resulted in the lowest intratumoral accumulation yield.

**Figure 8:**
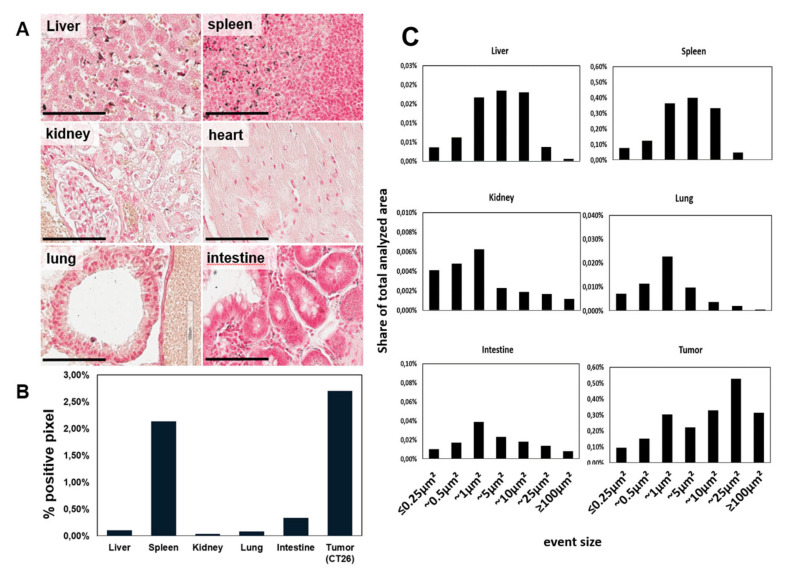
Biodistribution of cmHsp70.1-AuNPs: (**A**) Silver enhancement staining of liver, spleen, kidney, heart, lung, and intestine. Scale bar, 100 µm. (**B**) Pixel analysis of silver enhancement stainings in tumors and organs, given in positive pixel/mm^2^ tissue. (**C**) size distribution of silver enhanced cmHsp70.1-AuNP signals in different organs following i.v. injection.

To obtain the in vivo biodistribution characteristics of cmHsp70.1-AuNPs, 24 h following systemic application, we further analyzed silver enhanced sections of tumors, liver, spleen, kidneys, heart, lungs, and intestines of another group of tumor-bearing Balb/c mice (after subcutaneous and orthotopic injection) (Figure 8).

For an improved accuracy of the analysis, we used a machine-learning approach. The algorithm for the analysis of sections was trained on 28 slides in total and was applied for the analysis of 14 slides. In comparison to the organs, the highest accumulation of cmHsp70.1-AuNPs was found in the tumors, with 85.98 × 10^3^ ± 4.94 × 10^3^ positive pixel per mm^2^, which equals to 2.7% positive pixels per section (Figure 8B). Using the machine-learning approach, we found the majority of the nanoparticles accumulated in spleen (14.04 × 10^3^ ± 1.37 × 10^3^ pixel/mm^2^) (2.13% signal/section), liver (13.34 × 10^3^ ± 0.7 × 10^3^ pixel/mm^2^) (0.11% signal/section), and intestine (9.48 × 10^3^ ± 1.37 × 10^3^ pixel/mm^2^) (0.34% signal/section), followed by lungs (6.28 × 10^3^ ± 1.30 × 10^3^ pixel/mm^2^) (0.09% signal/section). In muscle, as represented by heart tissue, the accumulation of AuNPs was below the detection limit. Kidney was rarely affected, indicating an enterohepatic secretion of the AuNPs. In addition to the analysis of the overall entry of AuNPs in the organs and tumors, we utilized the machine-learning approach to analyze the size distribution of the nanoparticle agglumerations within the organs. The majority of positively stained events in liver and spleen resulted in a size range up to 10 µm. Accumulation of events in this size range might be suggested to be due to incorporation of the AuNP by macrophages and Kupffer cells, as indicated also in Figure 7B. The majority of AuNPs in kidney, lung, and intestine yielded in agglumerates of about 1 µm in size. In the tumor, cmHsp70.1-AuNP spots of 25 µm^2^ were dominant, followed by spots of 1 µm^2^ (Figure 8C). As tumor-associated macrophages exhibit large cytoplasmatic volumes, the accumulation of AuNPs above 10 µm^2^ suggest the appearance of this cell type. However, aggregates of 1 µm^2^ point to tumor cells with smaller cytoplasmic space as tumor cells. This finding is also supported by microscopical analysis of in vitro grown tumor cells with the majority of NP accumulating in cellular organelles of about 1 µm, following endocytosis (Figure 3).

## 3. Discussion

The discovery of suitable contrast agents for the visualization of tumors is one of the most important areas in clinical oncology. In tumor imaging, CT imaging is the most commonly used technique featuring fast scanning speed with high spatial resolution of a large portion of the body. Functional molecular imaging can be accomplished with positron-electron-tomography (PET), often used in combination with CT. However, certain limitations affect the quality of tumor visualization. PET imaging using ^18^F-FDG is highly dependent on a high tumor cell metabolism compared to the surrounding normal tissue. Furthermore, PET tracers need to exert ionizing effects, which in turn increases the patient’s risk to accumulate DNA mutations. An approach to overcome the limiting effects of PET/CT led to the development of spectral-CT analyzers [6,18], which use the k-edge discrimination of elements, allowing for their specific identification inside the body [19]. Another advantage of the spectral segmentation is the possibility to utilize multiple contrast agents within one imaging session, which reduces the X-ray expositions of the patient.

One further limitation of PET imaging is the spatial detection limit, leading to a potential miss of small lesions. Therefore, the usage of tumor-specific markers is beneficial.

Herein, the major stress-inducible member of the HSP70 family, Hsp70 (HspA1A), was used as a target for the tumor-specific uptake of functionalized AuNPs in different tumor entities. Hsp70 is expressed on the plasma membrane of a variety of tumor entities, whereas normal cells lack an Hsp70 membrane expression [9]. However, further studies investigate the role of membrane-associated Hsp70 in the context of inflammatory diseases, such as sepsis. It was found that membrane Hsp70 also plays a role in the regulation of inflammatory responses. A study of Hirsch et al. described polymorphonuclear neutrophils (PMNs), expressing mHsp70. These PMNs are recognized and lysed by γδ T-lymphocytes and therefore protect the host cells from inflammation-induced damage [20].

In previous studies, we observed that tumor cell membrane-associated Hsp70 is rapidly internalized and therefore mediates an efficient uptake of Hsp70-binding probes into the cytoplasm, such as fluorescence-labeled cmHsp70.1 antibody [15] and tumor-penetrating peptide (TPP) [21,22]. Since the binding epitope of cmHsp70.1 antibody is identical in mouse and human tumor cells [9], murine CT26 colon and mammary 4T1 tumor cell lines were used in the present study.

The application of AuNPs for imaging in vivo is a promising new approach in the field of in vivo imaging [7,23,24]. The uptake of antibody-conjugated nanoparticles into tumor cells is dependent on several factors: the expression density of receptors, which are expressed on the target cells; the distribution of the target epitopes throughout the cells [25]; the affinity of the tracer to the membrane epitope; and the speed of internalization [21,26].

In order to monitor the efficiency of functionalized AuNPs in vitro and in vivo, we applied different imaging modalities. Dynamic light scattering is a widely used technique to determine the hydrodynamic diameter of AuNPs in the nm–μm range.

Furthermore, the quantification of AuNPs within tumor cells is of relevance to estimate the concentration of nanoparticles that is necessary for noninvasive in vivo imaging of tumors as well as for their use as therapeutic agent. In our in vivo/ex vivo setup, we were able to detect aggregates of AuNPs in tumors and organs in perinuclear areas of around 1–2 μm in diameter as well as larger aggregates of around 25 µm in diameter. The presented machine-learning approach has proven to be beneficial for the analysis of the distribution of AuNPs. Next to calculating the quantity of gold signal within tumors and organs, we were able to separate the signal into groups, which allows for an easy analysis of AuNPs within different cell types. This is important for a more precise prediction of the biodistribution and possible toxic side effects of AuNPs.

Toxic side effects of AuNPs following i.v. application were investigated in previous studies [27,28]. No negative side effects such as loss in body weight or organic dysfunctions were observed in these experiments up to a concentration of 500 μg/mL [29]. Concordantly, in the in vivo experiments of this study, we did not observe any sign of toxicity at the injected concentration of 5 mg AuNPs per mouse. However, additional pharmacological and toxicological studies are needed to prove safety.

On the basis of the specific and quantitative uptake of cmHsp70.1-AuNPs in Hsp70-positive tumor cells and its imaging properties, our approach hints at a possible beneficial use in radiation therapy. Numerous studies have reported on the radiation-enhancing effect of AuNPs within tumor tissue [30,31].

First, promising findings for radiotherapy enhancement of AuNPs were achieved in preclinical mammary carcinoma studies [32]. The results of Hainfeld et al. showed an 86% 1-year survival for a combinatorial therapy of irradiation in presence of NPs compared to 20% for radiotherapy alone. Probable mechanisms involved in radiosensitization are, besides changes in the cell cycle or an elevated reactive oxygen species, the production and the release of secondary Auger electrons by gold in very close proximity to the nucleus [31]. In previous in vitro studies on the intracellular distribution of Hsp70 targeting AuNPs, we observed an increased accumulation of the NPs in close proximity to the nucleus 24 to 48 h after incubation [16], indicating possible beneficial effects of this approach for radiotherapeutical interventions. In summary, we demonstrate that the functionalization of AuNPs with cmHsp70.1 antibody is a highly promising approach for in vivo tumor targeting. Our preclinical studies show that the accumulation of AuNPs within the investigated tumors was sufficient for visualization by spectral-CT which allows 3D reconstructions and quantifications.

## 4. Materials and Methods

### 4.1. Antibody Coupling of AuNPs

Coupling of Hsp70-specific antibody (cmHsp70.1, multimmune, Germany) or an isotype-matched control IgG1 antibody (Sigma Aldrich, St. Luis, Mo, USA) to AuNPs (Nanopartz, Loveland, CO, USA) was done as described before [16]. Briefly, polyethylenglycol (PEG)-amine-coated spherical gold nanoparticles of 30 nm diameter were maleimide activated (Pierce, Thermo Fischer Scientific, Rockford, IL, USA) and incubated over night with sulfhydryl-activated antibodies. Antibody-coupled AuNPs or unconjugated AuNPs were analyzed and used for experiments within 24 h. Nanoparticles were analyzed (size, aggregation) by dynamic light scattering (DLS, Zetasizer NanoS, Malvern Instruments, Malvern, UK). Measurements were done in triplets and mean values were calculated.

### 4.2. Characterization of AuNPs

Antibody-conjugated AuNPs or unconjugated AuNPs were used for experiments directly after coupling. For AuNP characterization and controlling their aggregation, particle size was analyzed by dynamic light scattering (Zetasizer NanoS; Malvern Instruments, Malvern, UK). Only nanoparticles that produced single peaks were used for experiments. For analysis of the specific interaction of differentially functionalized AuNPs with Hsp70, 150 µg/mL nanoparticles dissolved in PBS were incubated with recombinant Hsp70 protein at a concentration of 0.5 µg/mL. Following a 4-h incubation time, the size of the clusters formed by IgG1-AuNP + Hsp70 and cmHsp70.1-AuNP + Hsp70, respectively, was analyzed by DLS.

### 4.3. Cell Culture

Murine colon carcinoma cell line CT26 (CT26.WT; American type culture collection (ATCC) #CRL-2638) and the mouse mammary carcinoma cell line 4T1 (ATCC #CRL-2539) were cultured in Roswell Park Memorial Institute 1640 medium supplemented with 10% (v/v) heat-inactivated fetal calf serum, 2 mM L-glutamine, 1 mM sodium pyruvate, and antibiotics (100 IU/mL penicillin and 100 μg/mL streptomycin). Cells were incubated at 37 °C in 95% humidity and 5% (v/v) CO_2_ and cultivated twice a week.

### 4.4. Assessment of Hsp70 Content of the Tumor Cells

The Hsp70 membrane phenotype of the cells was assessed by flow cytometry. Single cell suspensions of tumor cell lines were incubated with fluorescein-isothiocyanate (FITC)-conjugated cmHsp70.1 mAb for 30 min on ice. As controls, conjugation of an IgG1 isotype-matched antibody was done. After washing and adding propidium-iodide (PI) for life and dead discrimination, binding of antibodies was measured using a FACSCalibur instrument (BD Biosciences, Heidelberg, Germany). Data were analyzed using CellQuest Pro 6.0 software. Only PI-negative, viable cells were analyzed. To determine the membrane Hsp70 status of tumors grown in vivo, single cell suspension of freshly dissected tumors was generated by combined chopping and trypsin treatment of the tumors. Here, anti-mouse CD45 APC antibody was added to cmHsp70.1-FITC to distinguish tumor cells from mononuclear blood cells and infiltrated macrophages.

In-cell ELISA was performed, as described previously [21]. Briefly, cells grown in chamber slides were fixed with DAKO Fix & Perm kit (DAKO, Jena, Germany) and cellular membranes were permeabilized and incubated with cmHsp70.1-FITC antibody. Following microscopy with comparable settings, quantification of fluorescence signal was determined on the mean signal intensity values by ImageJ 1.52a image analysis software.

Quantification of the Hsp70 content was confirmed by an Hsp70 sandwich ELISA, as described elsewhere [33]. Shortly, after determination of the cell count, cells were lysed by incubation in Tris-HCl-based buffer containing 1% Triton-X100 and SDS. After centrifugation, the Hsp70 concentration in the supernatant was measured by total Hsp70 ELISA kit (R&D systems, Minneapolis, MN, USA) as described by the manufacturer. Each supernatant sample was measured in duplicates.

### 4.5. Animals

All animal procedures and their care were conducted in conformity with national and international guidelines (EU 2010/63) with approval from the local authorities of the Government of Upper Bavaria and ethical committee of Pavlov First Saint Petersburg State Medical University (St. Petersburg, Russia) (2015/068) and supervised by respective animal care and use committees. Animals were housed in standard animal rooms in individually ventilated cage systems (IVS Techniplast, Buggugiate, Italy) under specific pathogen-free conditions with free access to water and standard laboratory chow ad libitum. In total, 6 female Balb/c mice (aged 10–14 weeks, Charles River Laboratories, Sulzfeld, Germany) were used. Induction of subcutaneous tumors was done under inhalation anesthesia (1.8% isoflurane with medical O_2_) by injection of 5 × 10^5^ CT26 cells subcutaneously in the neck area or of 5 × 10^5^ 4T1 cells orthotopically in the 4th mammary fat pad. When tumors reached a size of 200 mm^3^, AuNPs were intravenously injected using standard procedures. In total, 5 mg unconjugated, IgG1-, or cmHsp70.1-AuNPs, suspended in phosphate buffered saline, were injected in a consecutive pattern of two times 2.5 mg per day within 48 h. Another 24 h after the second application, mice were euthanized under deep anesthesia, fixed in 4% neutral-buffered formalin for 5 days, and stored in 70% ethanol for further analysis.

### 4.6. Bright Field Microscopy

For light microscopy, cells were grown in 8-well chamber slides (NUNC-Nalgene; Thermo Fisher Scientific, Pittsburgh, PA, USA) at a concentration of 10,000 cells per well. Upon adherence, cells were incubated with AuNPs at a nontoxic concentration of 1 μg/mL. Cellular uptake of AuNPs or quantum dots of the same size were analyzed with a Zeiss Observer Z1 (Zeiss, Germany).

### 4.7. Transmission Electron Microscopy

Cells were co-incubated with functionalized and non-conjugated AuNPs (at a concentration 100 µg/mL) for 24 h. Following, incubation cells were washed with PBS, fixed for 1 h at 4 °C in 2.5% glutaraldehyde in 0.1 M cacodylate buffer (pH = 7.4), postfixed in 1% aqueous OsO4 (for 1 h), dehydrated, and embedded in Araldite-Epon mixture. Sections were assessed employing Zeiss Libra 120 electron microscope (Carl Zeiss, Germany).

### 4.8. Histology and Immunohistochemistry

Tumors and organs were either dissected following whole-body fixation of mice or fixed in 3.7% neutral-buffered formaldehyde and embedded in paraffin. Two-µm sections of the organs and tumors were prepared, and the morphology was visualized by standard H&E staining. For immunohistochemistry, the activity of the endogenous peroxidase was blocked with 1% hydrogen peroxide and 0.1% sodium azide. After antigen retrieval in citric acid buffer (pH 6) at 100 °C, sections were incubated with anti-Hsp70 antibody cmHsp70.1, followed by horse radish peroxidas (HRP)-labelled secondary rabbit anti-mouse antibody (Dako, Jena, Germany). Diaminobenzidine (Dako) was used as a chromogen. Sections were counterstained with 1% Mayer’s hematoxylin. To visualize the AuNPs by light microscopy, silver-enhancement staining was used according to the manufacturers’ protocol (Sigma-Aldrich, Darmstadt, Germany), followed by counterstaining of the nuclei with 0.1% Nuclear Fast Red solution (Morphisto, Frankfurt a.M., Germany). Slides were digitalized with a digital slide scanner (AT2, Leica, Wetzlar, Germany).

### 4.9. Spectral-CT Imaging and Image Acquisition

Spectral CT images were acquired at a Philips spectral CT scanner, as described before [18]. Briefly, a preclinical spectral photon-counting CT system (Philips Healthcare, Hamburg, Germany) was used to obtain axial scans over 360 ° at a beam voltage of 100 kVp. For optimal discrimination of the signals of AuNPs, a threshold was set at the k-edge energy of gold.

### 4.10. Spectral CT Image-Based Gold Quantification

Osirix^®^ MD v10.0.5 software (Pixmeo SARL, Bern, Switzerland) was used for analysis. Mean background was measured from several ROIs in the spleen. Gold amounts were calculated over the whole tumor volume (µg gold/mm^3^).

### 4.11. Deep Learning-Based Quantification of AuNP Histology

The biodistribution of silver-stained AuNPs in histological slides was assessed with the help of a deep neural network. Regions of interest in each slide were defined via manual delineation. Slides were subdivided into smaller, slightly overlapping patches of 1000 × 1000 pixels to meet memory constraints. In a first preliminary step, AuNP concentrations were segmented with the help of color-channel-specific dynamic thresholding and morphological operations (opening and closing with fixed kernels). Parameters were manually tuned to optimize results for the majority of slides. In a second step, the best resulting binary masks were manually selected and partially manually corrected. In a third step, a deep neural network was trained to segment AuNP concentrations on this basis. In a final step, the trained network was used to derive segmentations of AuNP concentrations for all slide patches. A k-fold rotation of data splits was applied so that the network yields segmentations on data samples that were not used for training, ensuring that the segmentation represents the result of the learned task rather than replicates the threshold-derived training data. This procedure yielded substantially more accurate and consistent segmentations of AuNP concentrations as compared to the preliminary thresholding procedure. Subsequent re-concatenation of slightly overlapping slide patches ruled out boundary effects and double counting. In a postprocessing step, all individual AuNP concentrations were identified via connected-component analysis, allowing to assess their individual sizes.

The deep neural network follows a U-net like architecture and consists of 4 levels of en- and decoding units. Each layer of encoding units doubles the number of feature channels, starting from 16 and ending at 265 at the deepest [16] level. The network was trained for 10 epochs on a training set of ca. 5000 patches. Details of architecture, implementation, and training procedure follow a previously described protocol [34].

## 5. Conclusions

Herein, we show that the visualization of tumor cells with AuNPs by addressing membrane Hsp70 is feasible. We present data showing a superior uptake of cmHsp70.1-AuNPs inside Hsp70 membrane-positive tumor cells. Inside the tumor cells, these particles accumulated in the perinuclear region within 24 h. The Hsp70 specificity was shown since unconjugated nanoparticles and nanoparticles conjugated with an irrelevant control antibody were not taken up into Hsp70 membrane-positive tumor cells. Furthermore, Hsp70 knockout tumor cells that do not express Hsp70 in the cytosol and on the plasma membrane showed no uptake of the cmHsp70.1-conjugated nanoparticles [16]. Quantification of the internalized cmHsp70.1-conjugated AuNPs reveals a high sensitivity for the detection of single cells. Experiments are ongoing to study the capability of cmHsp70.1-AuNPs for spectral CT imaging of further Hsp70 membrane-positive and negative tumor models and whether these NPs can be exploited for therapeutic approaches. In the future, these antibody-conjugated AuNPs might be useful for the diagnosis of tumors and for radiotherapeutic interventions.
